# Editorial Correspondence

**Published:** 1878-06

**Authors:** R. W. Westmoreland

**Affiliations:** Washington, Ark.


### EDITORIAL CORRESPONDENCE.

WASHINGTON, ARK., May 1, 1878.

*Editor Atlanta Medical and Surgical Journal:*

At the hazard of being accused of the *cacœthes scribendi* infection that is so distressing to observers, and is so difficult to cure, I once more offer my mite to the reading matter of your valuable JOURNAL.

More for the purpose of establishing a habit of communicating with the profession through the medium of the medical periodicals, than with any expectation of furnishing anything remarkably interesting, is my writing intended.

In recording my personal experience and observation, I may accidentally benefit some one who may be subjected to some of the individual exigencies that originated my own observations. It is in this manner of making known the practical experience of each practitioner, that facts exemplify the truths or expose the sophistries of theoretical premises.

There is no doubt that many there are who are deplorably affected with a proneness for accepting on *authority* that which should be illustrated by practical experience, and warp their minds into a conceited pedantry, and thereby partially establish a potent obstacle to the progress of rational medicine.

Such person will say, "Prof. Blank has said so-and-so, and as his field of observation, with his facilities for experience are much more extensive than mine, I consider him conclusive authority on this subject."

The fallacy of such a course is graphically illustrated in considering the revolution that has only very recently taken place in regard to the treatment of pneumonitis. On account of the failure to recognize the distinction between the *sthenic* and the asthenic varieties of the disease, success reported from one section as resulting from a scientific antiphlogistic course in treating the sthenic, led to a like active plan upon the asthenic, with, of course, disappointment. It is probable that we are nearing the dawn of such an epoch in the treatment of typhoid fever fever and cerebro-spinal meningitis. Various conflicting plans are suggested for their treatment, and varying reports as to the success in each plan may be noticed. Local circumstances and constitutional modifications, no doubt, exercise a decided influence, and exist as regulating elements in these affections. Many there are who will report almost universal success in the treatment of each, but such assertions are to be accepted either as ignorance on the part of the assertor, or as a sad commentary on the demagogism that is allowed in the profession. Such meretricious hypocrasies should be suppressed.

In such low, swampy regions as this, my seat of war, we meet the asthenic more often than the sthenic varieties of disease, and depressant remedies are almost totally discarded from our armamentarium. Though there are the appearances of a high grade of inflammation of the sthenic variety, yet from previous experience, reliance is had on the stimulant supporting plan of treatment, more than on the active anti-phlogistic.

As another effect of the malaria in rendering the system susceptible to disease, I have noticed uterine complaints are abundant in this locality, and are attributable principally to this cause.

Remedies that exert tonic effect upon the nervous centres and neutralize or expel the poison generally suffice. again resort must be had to uterine stimulants and hæmatinics with the addition occasionally of vaginal injections.

Out here we are exempt from the temptation that induces to that most nefarious of professional irregularity—accepting commission from drug stores for prescriptions made and directed to the store granting the subsidiary.

It is all very well to accept blanks and call lists, but when it comes to receiving a per cent. on each prescription sent, the principle of bribery is carried to its most criminal extent.

More anon.

R. W. Westmoreland, M.D.

